# The Multifaceted Function of Granzymes in Sepsis: Some Facts and a Lot to Discover

**DOI:** 10.3389/fimmu.2020.01054

**Published:** 2020-06-17

**Authors:** Marcela Garzón-Tituaña, Maykel A. Arias, José L. Sierra-Monzón, Elena Morte-Romea, Llipsy Santiago, Ariel Ramirez-Labrada, Luis Martinez-Lostao, José R. Paño-Pardo, Eva M. Galvez, Julián Pardo

**Affiliations:** ^1^Fundación Instituto de Investigación Sanitaria Aragón (IIS Aragón), Biomedical Research Centre of Aragón (CIBA), Zaragoza, Spain; ^2^Instituto de Carboquímica ICB-CSIC, Zaragoza, Spain; ^3^Hospital Clínico Universitario Lozano Blesa, Zaragoza, Spain; ^4^Nanotoxicology and Immunotoxicology Unit (UNATI), Fundación Instituto de Investigación Sanitaria Aragón (IIS Aragón), Zaragoza, Spain; ^5^Nanoscience Institute of Aragon (INA), University of Zaragoza, Zaragoza, Spain; ^6^Aragon I + D Foundation (ARAID), Zaragoza, Spain; ^7^Department of Biochemistry and Molecular and Cell Biology and Department of Microbiology, Preventive Medicine and Public Health, University of Zaragoza, Zaragoza, Spain; ^8^Centro de Investigación Biomédica en Red de Bioingeniería, Biomateriales y Nanomedicina (CIBER-BBN), Madrid, Spain

**Keywords:** sepsis, inflammatory cytokine, granzymes, coagulopathy, endothelial (dys)function, immunosuppression

## Abstract

Sepsis is a serious global health problem. In addition to a high incidence, this syndrome has a high mortality and is responsible for huge health expenditure. The pathophysiology of sepsis is very complex and it is not well-understood yet. However, it is widely accepted that the initial phase of sepsis is characterized by a hyperinflammatory response while the late phase is characterized by immunosuppression and immune anergy, increasing the risk of secondary infections. Granzymes (Gzms) are a family of serine proteases classified according to their cleavage specificity. Traditionally, it was assumed that all Gzms acted as cytotoxic proteases. However, recent evidence suggests that GzmB is the one with the greatest cytotoxic capacity, while the cytotoxicity of others such as GzmA and GzmK is not clear. Recent studies have found that GzmA, GzmB, GzmK, and GzmM act as pro-inflammatory mediators. Specially, solid evidences show that GzmA and GzmK function as extracellular proteases that regulate the inflammatory response irrespectively of its ability to induce cell death. Indeed, studies in animal models indicate that GzmA is involved in the cytokine release syndrome characteristic of sepsis. Moreover, the GZM family also could regulate other biological processes involved in sepsis pathophysiology like the coagulation cascade, platelet function, endothelial barrier permeability, and, in addition, could be involved in the immunosuppressive stage of sepsis. In this review, we provide a comprehensive overview on the contribution of these novel functions of Gzms to sepsis and the new therapeutic opportunities emerging from targeting these proteases for the treatment of this serious health problem.

## Introduction

The immune system is the most important line of defense against pathogens. The immune response to infection is initiated after the recognition of pathogen derived molecules known as Pathogen Associated Molecular Patterns (PAMPs) by specific receptors known as PAMP receptors. This process triggers the production of inflammatory, angiogenic, and chemotactic factors involved in the activation of innate and adaptive immune mechanisms leading to pathogen clearance, tissue repair, and resolution of the inflammatory response. However, if not properly regulated, inflammation may act as a double-edged sword that can trigger serious complications like sepsis. This syndrome is an important health problem, defined as “a life-threatening organ dysfunction caused by a dysregulated host response to infection” (1). Recent epidemiological studies estimate an annual incidence of 48.9 million sepsis cases with 11 million sepsis-related deaths representing 20% of all global deaths (2).

The pathophysiology of sepsis is very complex and it is not well-understood yet. Simultaneous and interrelated inflammatory and anti-inflammatory responses are developed (3, 4), although it is widely accepted that the initial phase of sepsis is characterized by a hyperinflammatory response while the late phase is characterized by immunosuppression and immune anergy that increases the risk of other opportunistic infections, specially bacterial and fungal. These opposite reactions hamper the development of effective treatments to reduce the inflammatory damage, and, at the same time, do not increase the risk or even help to prevent secondary infections. Indeed, most experimental trials to reduce the inflammatory response with general or specific anti-inflammatory molecules targeting PAMPs like endotoxin, PAMPRs like TLR4, complement, cytokines, or coagulation factors have not positively impacted on patient survival (5).

The reasons for the low efficacy of anti-inflammatory therapy are not clear since reduction of tissue damage and coagulation syndrome together with an effective antimicrobial therapy should be beneficial to improve disease outcome. One reason could be that anti-inflammatory therapy might reduce host pathogen clearance due to inhibition of inflammatory pathways important for the protective immune response against the infection responsible of sepsis (5). In addition, it is not clear yet whether the anti-inflammatory therapies tested up to now are able to prevent immunosuppression and anergy during sepsis. Thus, although molecules involved in exacerbated inflammation and coagulation like inflammatory cytokines (i.e., IL6, IL1, TNFα) have shown a good prognostic value for sepsis progression and severity, targets to regulate inflammation without compromising host immune response against the sepsis-inducing pathogen or against secondary infections have not been found yet. Recently, some members of a family of serine-proteases named granzymes (Gzms), that are expressed by immune and non-immune cells (6), have been found in extracellular human fluids of different inflammatory disorders like sepsis. In addition, they have been found to regulate the inflammatory response *in vitro* and *in vivo* during different inflammatory disorders related to infections like sepsis.

The granule exocytosis pathway is a specialized form of intracellular protein delivery by which lymphocytes release perforin and Gzms. Perforin exerts its action allowing the passage of granzymes into the cytosol of target cells to carry out their effector functions, including cytotoxic and non-cytotoxic functions (7). On the other hand, Gzms can be released into the extracellular milieu where they will exert some extracellular functions including regulation of inflammation, pathogen inactivation, or extracellular matrix remodeling (6, 8). Gzms are a family of serine proteases classified according to their cleavage specificity. Five Gzms in humans (A, B, H, K, and M) and 10 in mice (A, B, C, D, E, F, G, K, M, and N) have been described. GzmA and GzmB are the most abundant and best characterized (6). Traditionally, it was assumed that all Gzms acted as cytotoxic proteases. However, recent evidence suggests that intracellulary-delivered GzmB is the one with the greatest cytotoxic capacity, while the cytotoxicity of others such as GzmA and GzmK is in controversy (7, 9–13). A recent study has shown that GzmA might mediate pyroptotic cell death in human and mouse tumor cells (14), although the relevance of this finding needs to be confirmed in different experimental models, since it has been previously reported that the cytotoxic potential of intracellulary-delivered GzmA might differ between mouse and human (15). Regarding human GzmH, a few studies have shown that it can induce cell death and inactivate viral proteins (7). However, at present there is not any study correlating GzmH with the regulation of the inflammatory response or sepsis, and, thus, GzmH will not be further discussed in this review.

**Figure 1:**
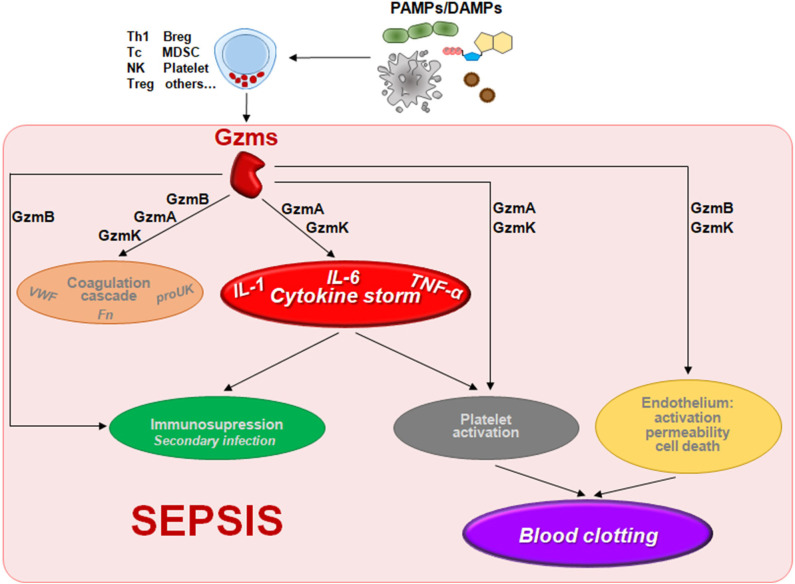
The potential contribution of the non-cytotoxic functions of granzymes to the pathophysiology of sepsis. Gzms can be released to the extracelular millieu by different cell types including NK cells, T cells, mast cells, platelets, or other cells stimulated by molecules presented in pathogens (fungal, bacterial, or viral PAMPs) or released by damaged cells (DAMPs) during sepsis. There different Gzms could contribute to different alterations found during sepsis like the inflammatory cytokine storm, blood clotting, cardiovascular disorders (coagulation and endotelial permeability), or immunosuppresion as described in the text. Abbreviations: Breg, regulatory B cell; MDSC, myeloid-derived suppressor cell; NK, natural killer; Tc, cytotoxic T cell; Th1, type 1 T helper cell; Treg, regulatory T cell; VWF, Von Willebrand factor; proUK, prourokinase; Fn, fibrin.

Recent studies have found that GzmA, GzmB, GzmK, and GzmM act as pro-inflammatory mediators and could be involved in the pathophysiology of sepsis (7, 10–12) (Table 1 and Figure 1). Thus, a detailed study of Gzms in different sepsis models as well as in patients undergoing different types of sepsis might provide new prognostic factors and therapeutic targets to overcome some of the limitations observed for other inflammation-related targets. This hypothesis is supported by different previous experimental findings indicating that deficiency in any single Gzm does not significantly reduce pathogen clearance, and, thus, their inhibition during sepsis would reduce inflammation without compromising host anti-pathogen immunity (6).

**Table 1:** Extracellular activity of granzymes on different cell types.

**Enzyme substrate specificity**	**Species**	**Cell type**	**Effect**	**Receptor involved**	**References**
GzmA Tryptase Lys, Arg	H	Monocytes	Induce expression of IL-6, IL-8, and TNF-α	–	(16)
		Monocytes	Induce expression of IL-1β, IL-6, and TNF-α	Inflammasomes/caspase-1	(17)
		Monocytes	Induce expression of IL-8 and MCP-1	Inflammasomes/caspase-1 TLR4	(18)
		Monocytes	Inactive gzmA potentiates the effect of LPS	TLR	(19)
		Lung Fibroblast	Induce expression of IL-6 and IL-8	–	(20)
		Intestinal Fibroblast	Induce expression of IL-6 and IL-8	–	
		Skin fibroblast	Induce expression of IL-8	–	
		Intestinal epithelial cells	Induce expression of IL-6 and IL-8	–	
	m	Neurons	Neurite retraction	PAR-1	(21)
		Macrophages	Induce expression of IL-1β	–	(17)
		Dendritic cells	Induce IFN-α and cell maturation	TLR-9	(22)
GzmB Aspartase Asp	H	Neurons	Neurotoxicity	PAR-1	(23)
		Smooth muscle cells	Cell death		(24)
	m	Endothelial cells	Cell death	–	(25)
		Endothelial cells	Disruptions of endothelial cell layer integrity by degrading proteins involved in tight junctions (Zonulin-1, PECAM, JAM, or cadherins)	–	(26)
GzmK Lys, Arg	H	Lung fibroblast	Induce the expression of IL-6, IL-8, and MCP-1	PAR-1	(27)
		Endothelial cells	Induce the expression of IL-6 and MCP-1	PAR-1	(28)
	m	Macrophages	Induce expression of IL-1β	–	(29)
GzmM^b^ Metase Met	H	Endothelial cells	Cleaves vWF and avoid plasma FVIII activation	–	(30)
GzmC-G Chymase Phe	m	–	–	–	
GzmH Chymase Phe	h	–	–	–	

*m, mouse; h, human; PAR, protease activated receptor; TLR, Toll like receptor*.

b *Extracellular GzmM cleaves vWF, releasing it from endothelial cell membrane and regulating its procoagulatory activity*.

In this review, we will present the available evidence in animal models and humans that suggests that Gzms might have a relevant role in sepsis pathophysiology. In addition, we will discuss the potential advantages of using Gzms as therapeutic targets to reduce organ damage without compromising pathogen clearance and to prevent immunosuppression-associated secondary infections. Since novel functions of Gzms, non-related with its ability to induce cell death, have emerged in the last years, we will focus on the potential impact of these non-cytotoxic functions in the pathophysiology of sepsis. Some of them are originated in *in vitro* studies in non-sepsis models. However, since several of these non-cytotoxic functions (explained in more detail in the following sections) form part of the pathophysiology of sepsis, we think that it will be useful to discuss its potential impact on sepsis, indicating when appropriate the needed to specifically confirm their relevance in sepsis.

## Granzymes are Elevated in Patients Suffering From Sepsis

Some studies have analyzed the presence of soluble Gzms in fluids from patients suffering from sepsis. These data show an increase in cellular and serum levels of some Gzms like GzmA, GzmB, and GzmK. Increased levels of GzmA and GzmB have been found in patients with severe gram negative bacterial infections as well as in healthy volunteers with an experimental endotoxemia with lipopolysaccharide (LPS) (31). GzmM and GzmK were also found in serum from volunteers with experimental endotoxemia (32) and GzmM was also elevated in serum from meningococcal sepsis (30). In contrast, GzmA levels were reduced in plasma during sepsis in burned patients (33). This result suggests that the level of GzmA in septic patients might depend on the underlying cause of sepsis. Supporting this hypothesis, Wensink et al. also showed that the release of GzmK and GzmM from human peripheral blood lymphocytes depended on the bacteria used for the stimulation (32). GzmK has also been found in the plasma of patients with sepsis as well as in bronchioalveolar fluid of patients with viral pneumonia (34, 35). However, the clinical significance of all these observations remains unknown.

A key question to understand the potential significance of soluble Gzms in sepsis is the regulation of its activity at the extracellular milieu. Like other proteases intracellular and extracellular Gzms might be tightly regulated to avoid unregulated proteolysis and cell/tissue damage. The main natural inhibitors that control Gzm activity are members of the Serpin (Serineprotease inhibitor) family. Different serpins have been found to inhibit extracellular Gzms like Inter-alpha-trypsin inhibitors (IaIs) (GzmA and GzmK), antithrombin III (GzmA), or bikunin (GzmK) (36). Thus, if extracellular Gzms are expected to play any role during sepsis it should be expected that they are not counteract by those inhibitors. Confirming this hypothesis and supporting a role for extracellular Gzms in sepsis it has been described that the levels of these inhibitors in plasma are reduced during sepsis. Indeed its concentration inversely correlated with mortality (37, 38).

Regarding the clinical utility of Gzms in sepsis prognosis, it was found that increased levels of GzmA and GzmB in CD8^+^T cells were associated with a worse prognosis in severe sepsis patients (39). Pending of validating the clinical significance of the presence of soluble Gzms in septic patients, studies in animal models *in vivo* indicate a key role for some Gzms like GzmA and GzmM in inflammation and sepsis. GzmA deficiency has been shown to protect against LPS-induced endotoxicosis (17, 40) as well as against sepsis induced by different bacterial pathogens including gram negative *Brucella microti* (41) or gram positive *Streptococcus pneumoniae* (42). These results suggest that the involvement of GzmA in sepsis is not related with Gram bacterial characteristics. Similarly, GzmM deficiency protects against LPS-induced endotoxicosis (40). In contrast, GzmB deficiency did not affect LPS-induced endotoxicosis (17, 40) or bacterial-induced sepsis (41). Importantly, increased survival in GzmA or GzmM deficient mice correlated with a reduction of the cytokine storm as measured by the levels of inflammatory cytokines in serum, organ damage, and coagulation activity confirming amelioration of the main sepsis pathological consequences. The course of sepsis in GzmK deficient mice remains to be elucidated.

The activity of Gzms during infectious diseases and sepsis seems to differ between anatomical site as well as the type of pathogen involved. For instance, a study showed that there was low level of GzmA in peritoneal lavage fluid of healthy mice while this level increased overtime during sepsis induced by *E. coli* (43). On the other hand, in a pneumonia-induced sepsis using a gram positive bacteria (*Streptococcus pneumoniae*) model the opposite was found (42). Here a small remark between the differences found in the pathophysiology of sepsis induced by gram negative and gram positive bacteria should be included. The onset and severity of sepsis may be defined by the molecular pathway involved in the activation of the immune system and the cytokines that can be induced in each case, both of which depend on the PAMPs expressed by the pathogen. Traditionally, the main responsible pathogens of sepsis have been considered gram negative bacteria. Nevertheless, it has been reported that gram positive bacteria are responsible for an ever-increasing number of septic events and have become the most frequent cause of sepsis, matching or even exceeding the sepsis caused by gram negative bacteria (44). It is known that the main receptors involved in the recognition of gram positive and negative bacteria are TLRs 2 and 4, respectively. This may be helpful for future studies and developments of sepsis treatments (44) and might help to explain the differences found regarding GzmA. For instance, a higher expression of IL-1β and IL-18 may indicate that the infection is due to a gram + bacteria while IL-6 and TNF would have a higher expression in gram negative infections (44). Indeed, GzmA has been found to produce very high levels of IL6 and TNF (Santiago et al. Cell Reports, Under review).

The inflammatory effect of GzmA does not seem to regulate bacterial control as GzmA deficient mice efficiently control infections like *Mycobacterium tuberculosis, Listeria monocytogenes, Brucella microti, Klebsiella pneumoniae, Streptococcus pneumoniae*, or *Escherichia coli* (6). Based on these experimental evidences it could be hypothesized that by targeting GzmA, bacteria-associated sepsis could be ameliorated without compromising the ability of the immune system to control infection. Thus, these findings open the opportunity to treat sepsis without causing immunosuppression and provide a new opportunity to overcome some of the limitations of other inflammatory targets unsuccessfully tested in clinical trials up to now.

## Which could be the Mechanisms Behind the Inflammatory Action of Gzms in Sepsis?

Several biological functions of Gzms that could contribute to the different alterations found in sepsis have been described. Mainly those related with the regulation of inflammatory responses, the coagulation cascade, and changes in vascular permeability (summarized in Figure 1). In this section we will describe the mechanisms activated by Gzms that could potentially contribute to the pathophysiology of sepsis with a special focus on the hyperinflammatory stage.

### Regulation of Inflammatory Cytokine Networks and Cytokine Storm

The hyperinflammatory response in sepsis is characterized by high levels of pro- and anti-inflammatory cytokines like IL-1β, IL-1α, IL-18, IL-17, TNFα, IL-6, MIF, HMGB1, IL-10, IL-4, and IL-13 (3, 45). This stage is also characterized by coagulation disorders, interstitial edema, hypotension, reduced perfusion, tissue hypoxia, mitochondrial dysfunction, and cell death (3, 4). It has been shown that extracellular GzmA is able to induce the production of different inflammatory cytokines such as IL-1β, TNF-α IL-6, or IL-8 in both mouse and human cells like monocytes, macrophages, fibroblasts, endothelial and epithelial cells (17, 20) (Table 1) which might contribute to the cytokine storm observed during sepsis. Indeed, most cytokines are reduced in GzmA deficient mice suffering from sepsis as indicated above. One of the first described substrates for GzmA is pro-interleukin 1β (pro IL-1 β), which after removal of the N-terminal part, generates the active inflammatory form IL-1β (46). However, direct cleavage of proIL1β and activation of IL1β has not been subsequently confirmed by different authors. Alternatively it has been reported that GzmA regulate the generation of inflammatory cytokines in human monocytes by caspase-1 and TLR4 dependent pathways (17, 18). Some other studies have reported that mouse GzmA is able to activate PAR1, a member of the Protease Activated Receptor (PAR) family, in mouse neurons (21). Since PARs are involved in platelet activation and thrombosis it could be suggested that PARs are involved in GzmA-mediated inflammation and coagulation alterations. However, as discussed below, the roles of PAR1 and 2 in sepsis are not clear yet and thus, *in vivo* extrapolation of all these results generated *in vitro* should be done with caution (47).

Alternatively, GzmA has been proposed to potentiate the effects of LPS on human monocytes by a mechanism dependent on TLR4 (19). Here authors showed that enzyme activity was not required for this effect, albeit the underlying mechanism was not clarified. This result contradicts previous findings that showed that human inactive GzmA was not able to induce the production of inflammatory cytokines in human monocytes (17). In addition, it has been shown that the inflammatory activity of mouse GzmA *in vivo* is significantly affected when GzmA inactivated with an inhibitor is used (48). The reasons for this discrepancy are not clear yet and will require further investigation.

GzmK displays a tryptase-like activity similar, but not identical (49, 50) to GzmA, and it might also induce the production of proinflammatory cytokines (Table 1). Nanomolar concentrations of GzmK induce the maturation and secretion of IL-1β suggesting that GzmK may augment GzmA-induced proinflammatory processes by differentially cleaving the same or different specific substrates (29). GzmK has also been found to activate PAR-1 in endothelial and fibroblast cells and to induce the generation of inflammatory cytokines (27, 28). However, as indicated above, its role in sepsis remains to be elucidated. Finally, as it has been previously discussed, GzmM is involved in endotoxicosis (40) although it is not known if GzmM is able to directly modulate inflammation on target cells (51).

### The Coagulation Cascade

Systemic inflammation activates coagulation and inhibits anticoagulant and fibrinolytic mechanisms which leads to a dysregulated procoagulant state (52). The tissue factor (TF), expressed in monocytes and microparticles from living or apoptotic cells, is the responsible of the coagulation cascade activating factor VII forming the FT/VIIa complex which activates complexes IX and X. All of these play an important role in platelet activation (53, 54). TF is the main initiator of *in vivo* coagulation and it is not expressed in circulation under healthy conditions. Nevertheless, under inflammatory conditions, TF is expressed in endothelial and fibroblast cells and released to the circulatory system due to the action of proinflammatory cytokines, C reactive protein, and final products of advanced glycation (55–57). GzmA and GzmK could contribute to enhanced activation of the coagulation cascade in sepsis by generating cytokines involved in endothelial cell activation and coagulation like TNF-α or IL6 (58–60). GzmB is able to enhance the proinflammatory activity of IL-1α by proteolytic cleavage (61), although as indicated above, GzmB deficiency does not protect from sepsis.

Gzms can also modulate the coagulation cascade irrespectively of its ability to induce inflammatory cytokines. It has been reported that GzmB and GzmM can degrade coagulation-related substrates such as Von Willebrand factor (VWF) (30) or fibrinogen (only GzmB) (62). VWF is a homeostatic plasma protein that promotes platelet adhesion. Furthermore, VWF stabilizes coagulation factor VIII (FVIII) in plasma protecting it from proteolytic degradation and prolonging FVIII half-life. In septic patients, increased levels of VWF have been reported, so this protein could be a link between inflammation and thrombosis (63). VWF is regulated by the metalloprotease ADAMTS13, which specifically cleaves VWF in its A2 domain (64). It has been reported that GzmB and GzmM could mimic the action of ADAMTS13 by cleaving VWF regulating its adhesive function and preventing its binding to FVIII although did not affect platelet aggregation (30, 62). If these processes are relevant *in vivo* during sepsis, GzmB and GzmM would be expected to attenuate coagulation, preventing systemic coagulation and organ failure. This would explain why GzmB deficiency does not protect from sepsis, but would not explain the increased resistance of GzmM deficient mice to endotoxicosis and the reduced coagulation activity observed in these mice. It should be expected that VWF lost procoagulatory activity after cleavage and, thus, GzmM would protect from systemic coagulation disorders. A similar paradox has been found for GzmA. GzmA has been found to activate pro-urokinase, an enzyme that presents anticoagulant activity (65). However, the detrimental role of GzmA in sepsis has been confirmed by several independent groups, suggesting that the *in vitro* function of Gzms in regulating proteins involved in coagulation might not be relevant during sepsis *in vivo*. However, this hypothesis should be clarified by analyzing specifically the role of Gzms in regulating the coagulation system *in vivo*.

### Regulation of Platelet Function

Platelets also play an important role in sepsis pathophysiology since the presence of thrombocytopenia is frequent in these patients. The role of platelets in sepsis is very complex as they can contribute to sepsis pathophysiology at different levels beyond regulation of haemostasis (66). Platelets can be directly activated by endotoxins, proinflammatory cytokines, or proteases such as thrombin (52, 67, 68). They contribute to the generation of inflammatory responses and, among other mediators, it has been shown that platelets can express GzmA and GzmB (18, 69, 70). Platelets can form clots on the endothelial damaged surface which produces elevated levels of VWF which is used as a marker for endothelial damage. It has been reported that the presence of proinflammatory cytokines such as IL-6, IL-8, and TNF-α (all of them induced by GzmA) are capable of induce the liberation of large VWF multimers that is a potent platelet aggregator. In septic patients, it has been observed elevated levels of VWF antigen which is related to a poor sickness prognosis (71). Here, as indicated above, since GzmB and GzmM have been found to cleave VWF and interfere with its procoagulant activity, it is tempting to speculate that these Gzms might have regulatory activities to prevent excessive VWF activity and clotting.

Curiously, GzmA was found to interact with the thrombin receptor (PAR1) in platelets and block thrombin-mediated responses (72). Although this interaction was not sufficient to induce *per se* platelet activation and aggregation, it might be possible that it can potentiate the effect of other ligands like endotoxin, a suggestion that has not been experimentally validated.

More recently it has been shown that during aging human platelets acquire GzmA expression which contributes to the generation of proinflammatory cytokines in monocytes (18). In light of these recent findings it will be interesting to analyse if Gzm expression is increased in older patients suffering from sepsis and its correlation with disease severity. Finally, as it will be discussed in the next section, it has been found that GzmB is expressed in platelets during sepsis using a mouse polymicrobial peritoneal sepsis model and might contribute to some of the alterations in vascular biology observed during sepsis (70).

### Endothelial Barrier Permeability: Any Role for Protease Activating Receptors (PARs)?

The fluid redistribution to the extravascular space observed during sepsis is a consequence of an increase in the endothelial permeability or of the barrier function loss. Previous studies suggest that the change in the membrane permeability is a consequence of the enzymatic cleavage of intercellular junction proteins, which results in a structural damage of endothelial cells. *In vivo* and *in vitro* studies have demonstrated that TNF-α is one of the responsible cytokines of this disorder and also that thrombin could potentiate them (73). Since some Gzms like GzmA and GzmM seem to be inflammatory mediators responsible of the detrimental effects of LPS in sepsis, including TNF-α production, they also could contribute to altered endothelial permeability.

The link between coagulation and inflammation has shown that the family of PARs play an important role because various proteases activated in the coagulation cascade will induce inflammation through PAR receptors. Activation of these receptors in endothelial cells critically contributes to the regulation of endothelial permeability. This family consists of four members, PAR-1,-2,-3,-4, that are expressed in cells present in the vasculature such as endothelial cells, monocytes, macrophages, platelets, fibroblasts and smooth muscle cells. PAR-1 is activated by thrombin, FXa (Activated factor X), trypsin and APC (Activated Protein C). It has also been reported that GzmA, B, and K are able to activate PAR1 in different cell types (21, 23, 27, 28). In contrast, PAR-2,-3 or−4 have not been found to be activated by any Gzm, albeit PAR2 participation in colitis has been indirectly proposed (74). Here it should be noted that neither activation of PAR2 by GzmA nor GzmA contribution to colitis were analyzed in the later study. Thus, it is not clear yet if PAR2 is activated by GzmA and the role of PAR2 in GzmA-mediated cytokine induction. A recent study found that a PAR-2 inhibitor (I-343) inhibited foot swelling induced by *in vivo* administration of GzmA (48). This result suggests that PAR-2 could be directly or indirectly involved in some of the biological functions of GzmA, but its specific role in the regulation of GzmA-mediated inflammatory cytokine production will require further experimental validation.

Despite the role of PAR-1 and 2 in the regulation of vascular biology and endothelial cell activation, its contribution to the pathophysiology of sepsis is complex and not completely clarified (47). Some data suggest that multiple activation of PAR receptors by coagulation proteases may contribute to inflammation in endotoxemia and in sepsis (75). However, it was found that PAR1 and/or PAR2 deficiency neither reduce the inflammatory response nor increase survival in a mouse model of endotoxemia, indicating that PAR1 and PAR2 are dispensable for LPS-induced sepsis (76, 77). In a later study, using PAR antagonists in wild type mice, it was shown that the role of PAR1 and PAR2 in sepsis is reversal, being detrimental in the early phase of sepsis and beneficial in later stages and thus, the net balance of PAR deficiency during sepsis was indistinguishable between wild type and PAR deficient mice (78). In contrast to PARs deficient mice, GzmA deficient mice are protected from endotoxemia and sepsis and, thus, PAR1 and 2 should not play an important role in GzmA-induced inflammation during sepsis.

PARs are expressed by a variety of cells and the function in each cell type might differ, which would explain the differences between the *in vitro* and the *in vivo* observations regarding PAR-1, GzmA, and sepsis. GzmA has been shown to activate PAR1 in neurons (21), but not in platelets (72) or monocytes (16). We have found that albeit a PAR-1 inhibitor, Vorapaxar, reduced IL6 production in mouse macrophages stimulated with GzmA, the protease was able to induce the same level of IL6 in macrophages from wt or PAR1 deficient mice (Santiago et al. Cell Reports, Accepted), indicating that PAR1 is dispensable for GzmA-induced inflammation in macrophages. This apparent contradictory result might be explained by the potential unspecific effects of Vorapaxar including toxicity (79). Whatever it is, it supports the previous findings suggesting that PAR1 is not activated by GzmA in platelets or monocytes (16, 72). In addition, it should be taken into account that even PAR1 could be activated in some cell types, its relative contribution to GzmA-mediated inflammation should be validated by using adequate inhibitors or genetic deficient cell models (KO or siRNA).

Regarding PAR-3 and−4, although they can be activated by thrombin and other proteases its role during sepsis has not been confirmed yet (80) and in addition there is not any evidence relating Gzms and them.

It seems that in light of the different results pointing to a complex regulation of PARs during sepsis, the involvement of PARs in the detrimental effects of GzmA on vascular permeability during sepsis will require further clarification and specific experimental validation.

### Endothelial Barrier Permeability: Extracellular Matrix Degradation, Matrikines and More

In addition to inflammatory cytokines and direct endothelial cell activation, other factors involved in the maintenance of vascular permeability could contribute to the loss of endothelial barrier function during sepsis. Some of these biological processes have been shown to be activated by Gzms *in vitro* like extracellular matrix (ECM) degradation, generation of products from ECM degradation with biological activity (matrikines) or killing of endothelial and smooth muscle cells (Table 1). Although the involvement of these processes in the mechanisms activated by Gzms during sepsis has not been directly analyzed, we would like to speculate on the potential implications of some of them.

Although GzmB deficiency does not increase survival during endotoxemia (40) or bacterial sepsis (41, 81), some of the biological functions of this protease might contribute to some of the vascular alterations observed in sepsis. For example, it has been shown that extracellular GzmB is able to directly kill cells involved in the maintenance of vascular architecture like endothelial and smooth muscle cells (25).

Gzm B Induces Smooth Muscle Cell Apoptosis in the Absence of Perforin. This process is based in its ability to induce ECM degradation and cell detachment (82, 83), which might activate cell death by anoikis (26, 82). Alternatively GzmB could disrupt endothelial cell barrier integrity by degrading proteins involved in tight junctions like Zonulin-1, PECAM, JAM, or Cadherins as previously shown (26). Later on it was found a role for GzmB in VE-cadherin cleavage and endothelial permeability *in vitro* and *in vivo* (84). In addition, ECM degradation by GzmB has been shown to release vascular endothelial growth factor which could affect vascular permeability (85). GzmB has also been found to affect wound healing, which was related to the ability of this protease to degrade fibronectin (86). Last but not least, it has been reported that GzmB may act on some of components of the EC involved in fibrillogenesis such as fibrillin-1 or decorin, increasing vascular permeability (87, 88), one of the most important pathological events that occur in sepsis.

Among other cell sources, platelets (70) and mast cells (26) have been found to express GzmB, but not perforin or GzmA, and thus, these cells could be the source of extracellular GzmB leading to its detrimental functions related with ECM degradation. In addition, it was found that GzmB of CD8^+^T and NK cells could modulate endothelial cell permeability and immune cells transmigration as a mechanism involved in host protection against viral infections (89). NK cells have been involved in sepsis (90, 91) and thus unregulated NK cells responses leading to a high release of GzmB due to PAMP-induced activation of NK cell receptors could transform a physiological protective mechanism into a pathological insult affecting endothelial cell permeability.

We would like to reiterate that the roles of GzmB in coagulation or in vascular permeability have not been studied yet in the context of a septic response and thus, all these hypotheses will require experimental validation.

Regarding GzmA, this protease can also degrade some proteins of the ECM like fibronectin or collagen IV (92, 93) and also could be released by NK cells during sepsis (41), although the biological functions of these processes remain unexplored.

## Granzymes at the Immunosuppressive Edge

With the improvement of intensive care services, the majority of patients with sepsis are able to survive the hyperinflammatory phase but enter a prolonged immunosuppression stage that has been called "immunoparalysis” in which they will be susceptible to secondary infections (94).

In the late stages of sepsis, murine studies reveal a relatively constant balance in proinflammatory and anti-inflammatory cytokines, although with a smaller magnitude when compared to the acute phase. One of the key features of immunosuppression in sepsis is the state of cellular anergy that can begin to occur even in the early stages of the disease (3, 95, 96). This immunosuppression stage is characterized by a decrease in antigen presentation, alteration in the expression of costimulatory molecules, and changes in lymphocytes populations. Here some experimental evidences acquired in different disease models suggest that some Gzms might also contribute to the different alterations leading to immunosuppression.

One of the causes of anergy is the decrease in antigen presentation and dendritic cell maturation leading to impaired protective cellular responses (97, 98). The function (expression for HLA-II and co-stimulatory molecules) and number of several antigen presenting cells has been found to be compromised in sepsis correlating with reduced survival like dendritic cells or monocytes (99–103). These alterations contribute to changes in the cytokine profiles and alterations in lymphocyte populations.

Although a direct impact of Gzms on antigen cell presentation has not been reported, GzmB might contribute to T cell immunosuppression by different means. All the hypotheses proposed below will require experimental validation in order to show if Gzms can contribute to the immunosuppressive stage in sepsis.

GzmB has been found to be expressed by human and mouse Treg cells (104, 105) and contribute to immunosuppression and cancer immune-evasion by inhibiting Tc and NK cell responses in tumor models (106) or to control viral-induced lung inflammation (107). Thus, it could be speculated that GzmB of Treg cell could contribute to the cellular immunosuppression observed in sepsis patients. T regulatory cells are known to contribute to immune homeostasis preventing reactions against self-healthy tissue and commensal microbiota. They inhibit the immune response at different levels like B, CD4 Th1, CD8 T cell, and NK cell activation or dendritic cell maturation and it has been found that sepsis patients present reduced numbers of CD4 Th1, CD8 T cell, and NK cell (108) and increased Treg cell number and activity (109–111), which contributes to secondary infection.

Alternatively, GzmB is able to cleave the zeta chain of the T cell antigen receptor (TCR) which renders un-functional T cells (112), being another potential mechanism by which GzmB could contribute to immunosuppression in septic patients.

More direct evidence on the role of Gzms in immunosuppression was recently provided by Freishtat et al., who reported that acute sepsis-induced alterations in the megakaryocyte-platelet transcriptional axis result in strongly cytotoxic platelets expressing GzmB. These platelets used GzmB to kill CD4^+^T cells contributing to lymphodepletion (69). Later on the same group reported that the mechanism of platelet-mediated GzmB-dependent lymphotoxicity required cell to cell contact and was perforin-independent (70).

## Conclusions and Future Perspectives

Sepsis is a global serious health problem for which early specific diagnosis and optimized treatment is crucial in order to reduce organ damage and improve survival. It is well-known the importance and influence of inflammation in the development and pathogenesis of sepsis (3, 5). However, the molecular bases to understand the dysregulated inflammatory response during sepsis are complex and often not well-understood. Indeed, most experimental trials to reduce inflammation during sepsis have not been successful and a positive impact on patient survival has not been reported. Among other reasons the impact of anti-inflammatory therapy on pathogen control by host immune response and the risk of secondary infections could contribute to the low efficacy observed during the trials with these therapies. During the last years, cumulative experimental evidences indicate that some members of the Gzm family, especially GzmA, have a key role in modulating inflammation and contribute to sepsis. Indeed, elevated levels of Gzms have been found in patients suffering from sepsis albeit the clinical significance remains unclear. In addition, *in vivo* mouse models show that GzmA deficiency protects from bacterial sepsis and endotoxemia indicating that GzmA have the potential to be used as biomarker and/or therapeutic target in sepsis (17, 40, 41). A current limitation of these studies is that the therapeutic potential of GzmA should be validated using a GzmA inhibitor in animal models expressing the protease. In addition, all *in vivo* studies have been performed using purified endotoxin or a single bacterial agent, and sepsis is often induced by more than a single microbial agent like peritoneal sepsis, one of the most common causes of sepsis. Moreover, the model of endotoxemia induced by LPS, albeit useful to study septic shock, does not provide information on the impact of regulation of inflammation on microbial infection.

Most studies in this field has been focused on the ability of some Gzms like GzmA, GzmK, and GzmM to induce the production of inflammatory cytokines in different cell types *in vitro* including monocytes, macrophages, endothelial cells, or fibroblasts. However, the role of GzmK *in vivo* has not been analyzed yet and GzmM has only been validated in the LPS-induced endotoxemia *in vivo* model. Apart from the ability to regulate the generation of the cytokine storm associated to the septic process and the consequences of this response to coagulation disorders and organ damage, *in vitro* evidences suggest that Gzms might also directly regulate other alterations found in sepsis like coagulation cascades, platelets function, or vascular permeability. Further experimental studies will be required to find out the relevance of these results in animal models *in vivo*, although it can be anticipated that it will not be easy to separate the effects due to Gzm-induced inflammatory cytokines from those that could be directly regulated by these proteases.

Apart from the disorders related with the inflammatory response leading to disseminated coagulation and organ damage, as discussed above, Gzms might also contribute to the immunosuppressive stage commonly observed in septic patients, responsible for a high morbidity/mortality due to secondary infections. Indirect evidences from other experimental models suggest that some Gzms might contribute to immunosuppression and, thus, it might be worth to dedicate some efforts to find out the role of Gzms in promoting immunosuppression and/or anergy during sepsis.

Despite the limitations mentioned above and all the hypotheses and speculations pending of experimental validation, some Gzms like GzmA or GzmK might present an important advantage in comparison with other inflammatory molecules that have been proposed and tested as therapeutic targets. GzmA or GzmK deficiency does not predispose to infection and these animals are able to efficiently clear most experimental infections (6, 113). Thus, targeting Gzms might reduce sepsis pathology without compromising the host immune response against the offending pathogen/s and, thus, enhancing the chances of efficient antimicrobial treatment to re-establish immune homeostasis and reduce immune-associated damage. Before these therapies can be developed and tested in potential clinical trials, it will be required to better understand the biology of granzymes during sepsis and the mechanisms involved.

## Author Contributions

MG-T, MA, and JP-P designed and wrote the first draft. LM-L prepared Figure 1. All authors wrote and revised the manuscript.

### Conflict of Interest

The authors declare that the research was conducted in the absence of any commercial or financial relationships that could be construed as a potential conflict of interest.
